# Does time management work? A meta-analysis

**DOI:** 10.1371/journal.pone.0245066

**Published:** 2021-01-11

**Authors:** Brad Aeon, Aïda Faber, Alexandra Panaccio

**Affiliations:** 1 Concordia University, Sir George Williams Campus, Montreal, Quebec, Canada; 2 FSA Ulaval, Laval University, Quebec City, Quebec, Canada; Universidad Nacional de Educacion a Distancia (UNED), SPAIN

## Abstract

Does time management work? We conducted a meta-analysis to assess the impact of time management on performance and well-being. Results show that time management is moderately related to job performance, academic achievement, and wellbeing. Time management also shows a moderate, negative relationship with distress. Interestingly, individual differences and contextual factors have a much weaker association with time management, with the notable exception of conscientiousness. The extremely weak correlation with gender was unexpected: women seem to manage time better than men, but the difference is very slight. Further, we found that the link between time management and job performance seems to increase over the years: time management is more likely to get people a positive performance review at work today than in the early 1990s. The link between time management and gender, too, seems to intensify: women’s time management scores have been on the rise for the past few decades. We also note that time management seems to enhance wellbeing—in particular, life satisfaction—to a greater extent than it does performance. This challenges the common perception that time management first and foremost enhances work performance, and that wellbeing is simply a byproduct.

## Introduction

Stand-up comedian George Carlin once quipped that in the future a “time machine will be built, but no one will have time to use it” [1]. Portentously, booksellers now carry one-minute bedtime stories for time-starved parents [2] and people increasingly speed-watch videos and speed-listen to audio books [3–5]. These behaviors are symptomatic of an increasingly harried society suffering from chronic time poverty [6]. Work is intensifying—in 1965 about 50% of workers took breaks; in 2003, less than 2% [7]. Leisure, too, is intensifying: people strive to consume music, social media, vacations, and other leisure activities ever more efficiently [8–11].

In this frantic context, time management is often touted as a panacea for time pressure. Media outlets routinely extol the virtues of time management. Employers, educators, parents, and politicians exhort employees, students, children, and citizens to embrace more efficient ways to use time [12–16]. In light of this, it is not surprising that from 1960 to 2008 the frequency of books mentioning time management shot up by more than 2,700% [17].

Time management is defined as “a form of decision making used by individuals to structure, protect, and adapt their time to changing conditions” [18]. This means time management, as it is generally portrayed in the literature, comprises three components: structuring, protecting, and adapting time. Well-established time management measures reflect these concepts. Structuring time, for instance, is captured in such items as “Do you have a daily routine which you follow?” and “Do your main activities during the day fit together in a structured way?” [19]. Protecting time is reflected in items such as “Do you often find yourself doing things which interfere with your schoolwork simply because you hate to say ‘No’ to people?” [20]. And adapting time to changing conditions is seen in such items as “Uses waiting time” and “Evaluates daily schedule” [21].

Research has, furthermore, addressed several important aspects of time management, such as its relationship with work-life balance [22], whether gender differences in time management ability develop in early childhood [23], and whether organizations that encourage employees to manage their time experience less stress and turnover [24]. Despite the phenomenal popularity of this topic, however, academic research has yet to address some fundamental questions [25–27].

A critical gap in time management research is the question of whether time management works [28, 29]. For instance, studies on the relationship between time management and job performance reveal mixed findings [30, 31]. Furthermore, scholars’ attempts to synthesize the literature have so far been qualitative, precluding a quantitative overall assessment [18, 32, 33]. To tackle this gap in our understanding of time management, we conducted a meta-analysis. In addressing the question of whether time management works, we first clarify the criteria for effectiveness. In line with previous reviews, we find that virtually all studies focus on two broad outcomes: performance and wellbeing [32].

Overall, results suggest that time management enhances job performance, academic achievement, and wellbeing. Interestingly, individual differences (e.g., gender, age) and contextual factors (e.g., job autonomy, workload) were much less related to time management ability, with the notable exception of personality and, in particular, conscientiousness. Furthermore, the link between time management and job performance seems to grow stronger over the years, perhaps reflecting the growing need to manage time in increasingly autonomous and flexible jobs [34–37].

Overall, our findings provide academics, policymakers, and the general audience with better information to assess the value of time management. This information is all the more useful amid the growing doubts about the effectiveness of time management [38]. We elaborate on the contributions and implications of our findings in the discussion section.

## What does it mean to say that time management works?

In the din of current debates over productivity, reduced workweeks, and flexible hours, time management comes to the fore as a major talking point. Given its popularity, it would seem rather pointless to question its effectiveness. Indeed, time management’s effectiveness is often taken for granted, presumably because time management offers a seemingly logical solution to a lifestyle that increasingly requires coordination and prioritization skills [39, 40].

Yet, popular media outlets increasingly voice concern and frustration over time management, reflecting at least part of the population’s growing disenchantment [38]. This questioning of time management practices is becoming more common among academics as well [41]. As some have noted, the issue is not just whether time management works. Rather, the question is whether the techniques championed by time management gurus can be actually counterproductive or even harmful [26, 42]. Other scholars have raised concerns that time management may foster an individualistic, quantitative, profit-oriented view of time that perpetuates social inequalities [43, 44]. For instance, time management manuals beguile readers with promises of boundless productivity that may not be accessible to women, whose disproportionate share in care work, such as tending to young children, may not fit with typically male-oriented time management advice [45]. Similarly, bestselling time management books at times offer advice that reinforce global inequities. Some manuals, for instance, recommend delegating trivial tasks to private virtual assistants, who often work out of developing countries for measly wages [46]. Furthermore, time management manuals often ascribe a financial value to time—the most famous time management adage is that time is money. But recent studies show that thinking of time as money leads to a slew of negative outcomes, including time pressure, stress, impatience, inability to enjoy the moment, unwillingness to help others, and less concern with the environment [47–51]. What’s more, the pressure induced by thinking of time as money may ultimately undermine psychological and physical health [52].

Concerns over ethics and safety notwithstanding, a more prosaic question researchers have grappled with is whether time management works. Countless general-audience books and training programs have claimed that time management improves people’s lives in many ways, such as boosting performance at work [53–55]. Initial academic forays into addressing this question challenged those claims: time management didn’t seem to improve job performance [29, 30]. Studies used a variety of research approaches, running the gamut from lab experiments, field experiments, longitudinal studies, and cross-sectional surveys to experience sampling [28, 56–58]. Such studies occasionally did find an association between time management and performance, but only in highly motivated workers [59]; instances establishing a more straightforward link with performance were comparatively rare [31]. Summarizing these insights, reviews of the literature concluded that the link between time management and job performance is unclear; the link with wellbeing, however, seemed more compelling although not conclusive [18, 32].

It is interesting to note that scholars often assess the effectiveness time management by its ability to influence some aspect of performance, wellbeing, or both. In other words, the question of whether time management works comes down to asking whether time management influences performance and wellbeing. The link between time management and performance at work can be traced historically to scientific management [60]. Nevertheless, even though modern time management can be traced to scientific management in male-dominated work settings, a feminist reading of time management history reveals that our modern idea of time management also descends from female time management thinkers of the same era, such as Lillian Gilbreth, who wrote treatises on efficient household management [43, 61, 62]. As the link between work output and time efficiency became clearer, industrialists went to great lengths to encourage workers to use their time more rationally [63–65]. Over time, people have internalized a duty to be productive and now see time management as a personal responsibility at work [43, 66, 67]. The link between time management and academic performance can be traced to schools’ historical emphasis on punctuality and timeliness. In more recent decades, however, homework expectations have soared [68] and parents, especially well-educated ones, have been spending more time preparing children for increasingly competitive college admissions [69, 70]. In this context, time management is seen as a necessary skill for students to thrive in an increasingly cut-throat academic world. Finally, the link between time management and wellbeing harks back to ancient scholars, who emphasized that organizing one’s time was necessary to a life well-lived [71, 72]. More recently, empirical studies in the 1980s examined the effect of time management on depressive symptoms that often plague unemployed people [19, 73]. Subsequent studies surmised that the effective use of time might prevent a host of ills, such as work-life conflict and job stress [22, 74].

Overall, then, various studies have looked into the effectiveness of time management. Yet, individual studies remain narrow in scope and reviews of the literature offer only a qualitative—and often inconclusive—assessment. To provide a more quantifiable answer to the question of whether time management works, we performed a meta-analysis, the methods of which we outline in what follows.

## Method

### Literature search and inclusion criteria

We performed a comprehensive search using the keywords “time management” across the EBSCO databases *Academic Search Complete*, *Business Source Complete*, *Computers & Applied Sciences Complete*, *Gender Studies Database*, *MEDLINE*, *Psychology and Behavioral Sciences Collection*, *PsycINFO*, *SocINDEX*, and *Education Source*. The search had no restrictions regarding country and year of publication and included peer-reviewed articles up to 2019. To enhance comprehensiveness, we also ran a forward search on the three main time management measures: the Time Management Behavior Scale [21], the Time Structure Questionnaire [19], and the Time Management Questionnaire [20]. (A forward search tracks all the papers that have cited a particular work. In our case the forward search located all the papers citing the three time management scales available on *Web of Science*.)

Time management measures typically capture three aspects of time management: structuring, protecting, and adapting time to changing conditions. Structuring refers to how people map their activities to time using a schedule, a planner, or other devices that represent time in a systematic way [75–77]. Protecting refers to how people set boundaries around their time to repel intruders [78, 79]. Examples include people saying no to time-consuming requests from colleagues or friends as well as turning off one’s work phone during family dinners. Finally, adapting one’s time to changing conditions means, simply put, to be responsive and flexible with one’s time structure [80, 81]. Furthermore, time management measures typically probe behaviors related to these three dimensions (e.g., using a schedule to structure one’s day, making use of downtime), although they sometimes also capture people’s attitudes (e.g., whether people feel in control of their time).

**Fig 1:**
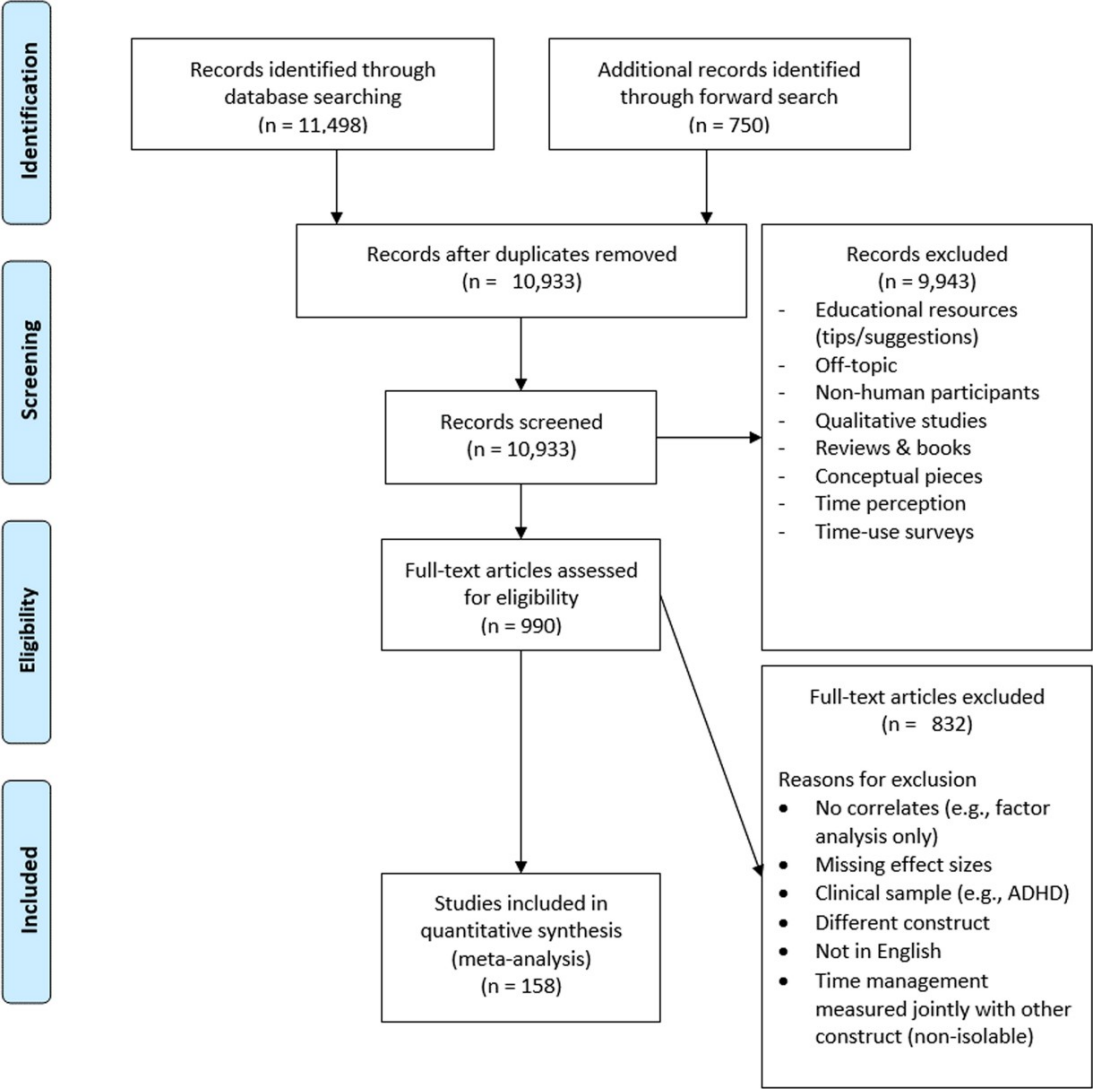
PRISMA chart summarizing the screening process [82].

As shown in Fig 1, the initial search yielded 10,933 hits, excluding duplicates.

The search included no terms other than “time management” to afford the broadest possible coverage of time management correlates. Nevertheless, as shown in Table 1, we focused exclusively on quantitative, empirical studies of time management in non-clinical samples. Successive rounds of screening, first by assessing paper titles and abstracts and then by perusing full-text articles, whittled down the number of eligible studies to 158 (see Fig 1).

**Table 1:** Summary of inclusion and exclusion criteria.

Inclusion Criteria	Exclusion Criteria
Study must contain a quantitative measure of time management (e.g., scale, survey, questionnaire) and/or feature a time management experiment with at least one control group	Qualitative approaches (e.g., interviews, case studies)
Construct must be related to time management, such as time structure, time planning, scheduling, time management behaviors, time management practice, time management skills, and attitudes toward time management	Time-use studies (e.g., national time-use surveys, individual-level time-tracking studies), time perception studies, studies on non-personal time management (e.g., real-time management in supply chains), and time management studies focusing mainly on clinical samples (e.g., with chronic pain or ADHD)
Study must be about time management in relation to other variables (e.g., life satisfaction, stress, academic achievement)	Studies focusing exclusively on time management (e.g., factor analyses)

### Data extraction and coding

We extracted eligible effect sizes from the final pool of studies; effect sizes were mostly based on means and correlations. In our initial data extraction, we coded time management correlates using the exact variable names found in each paper. For instance, “work-life imbalance” was initially coded in those exact terms, rather than “work-life conflict.” Virtually all time management correlates we extracted fell under the category of performance and/or wellbeing. This pattern tallies with previous reviews of the literature [18, 32]. A sizable number of variables also fell under the category of individual differences and contextual factors, such as age, personality, and job autonomy. After careful assessment of the extracted variables, we developed a coding scheme using a nested structure shown in Table 2.

**Table 2:** Coding scheme for time management correlates.

Performance				Wellbeing		Individual Differences			
Professional Setting		Academic Setting		Positive (wellbeing)	Negative (distress)	Demographics	Personality	Attributes and Attitudes	Contextual Factors
**Results-based**	**Behavior-based**	**Results-based**	**Behavior-based**	Job Satisfaction	Emotional Exhaustion	Age	Agreeableness	Internal Locus of Control	Job Autonomy
Job performance	Creativity	GPA	Procrastination (reverse coded)	Life Satisfaction	Stress	Gender	Extraversion	Type A	Role Overload
	Helping Behavior	Standardized Tests	Motivation	Mental Health (positive)	Work-life Conflict	Education	Conscientiousness	Self-esteem	Time Management Training
	Job Involvement	Test Scores		Optimism	Anxiety	Number of Children	Neuroticism	Protestant Work Ethic	
	Procrastination (reverse coded)			Physical health (positive)	Depression	Marital Status	Openness	Multitasking	
	Motivation			Positive affect	Psychological Distress			Cognitive Ability	
	Proactiveness			Self-actualization	Hopelessness			Hours Worked	
				Sense of purpose	Boredom				
				Wellbeing	Negative Affect				
					Worry				
					Physical Distress				

Aeon and Aguinis suggested that time management influences performance, although the strength of that relationship may depend on how performance is defined [18]. Specifically, they proposed that time management may have a stronger impact on behaviors conducive to performance (e.g., motivation, proactiveness) compared to assessments of performance (e.g., supervisor rankings). For this reason, we distinguish between results- and behavior-based performance in our coding scheme, both in professional and academic settings. Furthermore, wellbeing indicators can be positive (e.g., life satisfaction) or negative (e.g., anxiety). We expect time management to influence these variables in opposite ways; it would thus make little sense to analyze them jointly. Accordingly, we differentiate between wellbeing (positive) and distress (negative).

In our second round of coding, we used the scheme shown in Table 2 to cluster together kindred variables. For instance, we grouped “work-life imbalance,” “work-life conflict” and “work-family conflict” under an overarching “work-life conflict” category. The authors reviewed each variable code and resolved rare discrepancies to ultimately agree on all coded variables. Note that certain variables, such as self-actualization, covered only one study (i.e., one effect size). While one or two effect sizes is not enough to conduct a meta-analysis, they can nonetheless be grouped with other effect sizes belonging to the same category (e.g., self-actualization and sense of purpose belong the broader category of overall wellbeing). For this reason, we included variables with one or two effect sizes for comprehensiveness.

### Meta-analytic procedures

We conducted all meta-analyses following the variables and cluster of variables outlined in Table 2. We opted to run all analyses with a random effects model. The alternative—a fixed effects model—assumes that all studies share a common true effect size (i.e., linking time management and a given outcome) which they approximate. This assumption is unrealistic because it implies that the factors influencing the effect size are the same in all studies [83]. In other words, a fixed effects model assumes that the factors affecting time management are similar across all studies—the fallacy underlying this assumption was the main theme of Aeon and Aguinis’s review [18]. To perform our analyses, we used Comprehensive Meta-Analysis v.3 [84], a program considered highly reliable and valid in various systematic assessments [85, 86].

Meta-analyses do not typically perform calculations on correlations (e.g., Pearson’s r). Instead, we transformed correlations into Fisher’s z scales [83]. The transformation was done with $z=0.5\times \ln\left(\frac{1+r}{1-r}\right)$, where *r* represents the correlation extracted from each individual study. The variance of Fisher’s Z was calculated as $V_{z}=\frac{1}{n-3}$ where *n* corresponds to the study’s sample size; the standard error of Fisher’s Z was calculated as ${SE}_{z}=\sqrt{V_{z}}$.

In many cases, studies reported how variables correlated with an overall time management score. In some cases, however, studies reported only correlations with discrete time management subscales (e.g., short-range planning, attitudes toward time, use of time management tools), leaving out the overall effect. In such cases, we averaged out the effect sizes of the subscales to compute a summary effect [83]. This was necessary not only because meta-analyses admit only one effect size per study, but also because our focus is on time management as a whole rather than on subscales. Similarly, when we analyzed the link between time management and a high-level cluster of variables (e.g., *overall* wellbeing rather than specific variables such as life satisfaction), there were studies with more than one relevant outcome (e.g., a study that captured both life satisfaction and job satisfaction). Again, because meta-analyses allow for only one effect size (i.e., variable) per study, we used the mean of different variables to compute an overall effect sizes in studies that featured more than one outcome [83].

## Results

### Overall description of the literature

We analyzed 158 studies for a total number of 490 effect sizes. 21 studies explored performance in a professional context, 76 performance in an academic context, 30 investigated wellbeing (positive), and 58 distress. Interestingly, studies did not systematically report individual differences, as evidenced by the fact that only 21 studies reported correlations with age, and only between 10 and 15 studies measured personality (depending on the personality trait). Studies that measured contextual factors were fewer still—between 3 and 7 (depending on the contextual factor). These figures fit with Aeon and Aguinis’s observation that the time management literature often overlooks internal and external factors that can influence the way people manage time [18].

With one exception, we found no papers fitting our inclusion criteria before the mid-1980s. Publication trends also indicate an uptick in time management studies around the turn of the millennium, with an even higher number around the 2010s. This trend is consistent with the one Shipp and Cole identified, revealing a surge in time-related papers in organizational behavior around the end of the 1980s [87].

It is also interesting to note that the first modern time management books came out in the early 1970s, including the *The Time Trap* (1972), by Alec MacKenzie and *How to Get Control of your Time and your Life* (1973), by Alan Lakein. These books inspired early modern time management research [21, 58, 88]. It is thus very likely that the impetus for modern time management research came from popular practitioner manuals.

To assess potential bias in our sample of studies, we computed different estimates of publication bias (see Table 3). Overall, publication bias remains relatively low (see funnel plots in S1). Publication bias occurs when there is a bias against nonsignificant or even negative results because such results are seen as unsurprising and not counterintuitive. In this case, however, the fact that time management is generally expected to lead to positive outcomes offers an incentive to publish nonsignificant or negative results, which would be counterintuitive [89]. By the same token, the fact that some people feel that time management is ineffective [38] provides an incentive to publish papers that link time management with positive outcomes. In other words, opposite social expectations surrounding time management might reduce publication bias.

**Table 3:** Publication bias estimates for each time management outcome.

	Job performance	Academic achievement	Wellbeing	Distress
**Classic Fail-Safe *N***	344	2,735	6,496	9,333
**Orwin’s Fail-Safe *N***	75	309	339	364
**Egger’s Test of the Intercept**	B(0) = 2.76	B(0) = 1.18	B(0) = 0.31	B(0) = -1.18
	CI (95%) = (-.77; 6.28)	CI (95%) = (-.36; 2.72)	CI (95%) = (-.4.08; 4.69)	CI (95%) = (-.3.31; 0.94)
	*p* > .05	*p* > .05	*p* > .05	*p* > .05
**Duval & Tweedie’s Trim and Fill Method**	1 study missing	0 studies missing	0 studies missing	14 studies missing
	New effect size = .188			New effect size = .283
**Overall Degree of Publication Bias**	**Moderate**	**Low**	**Low**	**Moderate**

Finally, we note that the link between time management and virtually all outcomes studied is highly heterogeneous (as measured, for instance, by Cochran’s *Q* and Higgins & Thompson’s *I*^*2*^; see tables below). This high level of heterogeneity suggests that future research should pay more attention to moderating factors (e.g., individual differences).

### Time management and performance in professional settings

Overall, time management has a moderate impact on performance at work, with correlations hovering around *r* = .25. We distinguish between results-based and behavior-based performance. The former measures performance as an outcome (e.g., performance appraisals by supervisors) whereas the latter measures performance as behavioral contributions (e.g., motivation, job involvement). Time management seems related to both types of performance. Although the effect size for results-based performance is lower than that of behavior-based performance, moderation analysis reveals the difference is not significant (p > .05), challenging Aeon and Aguinis’s conclusions [18].

Interestingly, the link between time management and performance displays much less heterogeneity (see *Q* and *I*^*2*^ statistics in Table 4) than the link between time management and other outcomes (see tables below). The studies we summarize in Table 4 include both experimental and non-experimental designs; they also use different time management measures. As such, we can discount, to a certain extent, the effect of methodological diversity. We can perhaps explain the lower heterogeneity by the fact that when people hold a full-time job, they usually are at a relatively stable stage in life. In school, by contrast, a constellation of factors (e.g., financial stability and marital status, to name a few) conspire to affect time management outcomes. Furthermore, work contexts are a typically more closed system than life in general. For this reason, fewer factors stand to disrupt the link between time management and job performance than that between time management and, say, life satisfaction. Corroborating this, note how, in Table 6 below, the link between time management and *job* satisfaction (*I*^*2*^ = 58.70) is much less heterogeneous than the one between time management and *life* satisfaction (*I*^*2*^ = 95.45).

**Table 4:** Time management and performance in professional settings.

Variable	k	N	r	95% CI	Q(df)	*τ*^2^	*τ*^2^(SE)	*I*^*2*^
**Performance (overall)**	21	3,990	0.259***	0.197–0.318	77.32 (20)	0.016	0.007	74.13
** Results-based performance (overall)**	13	2,532	0.221***	0.144–0.295	44.19 (12)	0.015	0.009	72.84
** Behavior-based performance (overall)**	13	2,474	0.297***	0.225–0.365	40.56 (12)	0.013	0.008	70.41
Creativity	1	213	0.460***	0.347–0.560	-	-	-	-
Helping behavior	1	254	0.160*	0.038–0.278	-	-	-	-
Job involvement	4	617	0.207***	0.129–0.282	2.99 (3)	0	0.006	0
Procrastination (reverse coded)	2	198	0.374**	0.166–0.550	1.61 (1)	0.012	0.046	37.92
Motivation	4	711	0.352***	0.226–0.467	10.12 (3)	0.014	0.016	70.37
Proactiveness	3	813	0.267***	0.121–0.401	8.81 (2)	0.014	0.018	77.30

* p < .05

** p < .01

*** p < .001.

k = number of studies related to the variable | N = total sample size related to the variable.

r = effect size of the correlation between time management and the variable | 95% CI = confidence interval of the effect size.

Q = Cochran’s Q, a measure of between-study heterogeneity | *τ*^2^ = measure of between-study variance | *I*^*2*^ = alternative measure of between-study heterogeneity.

**Fig 2:**
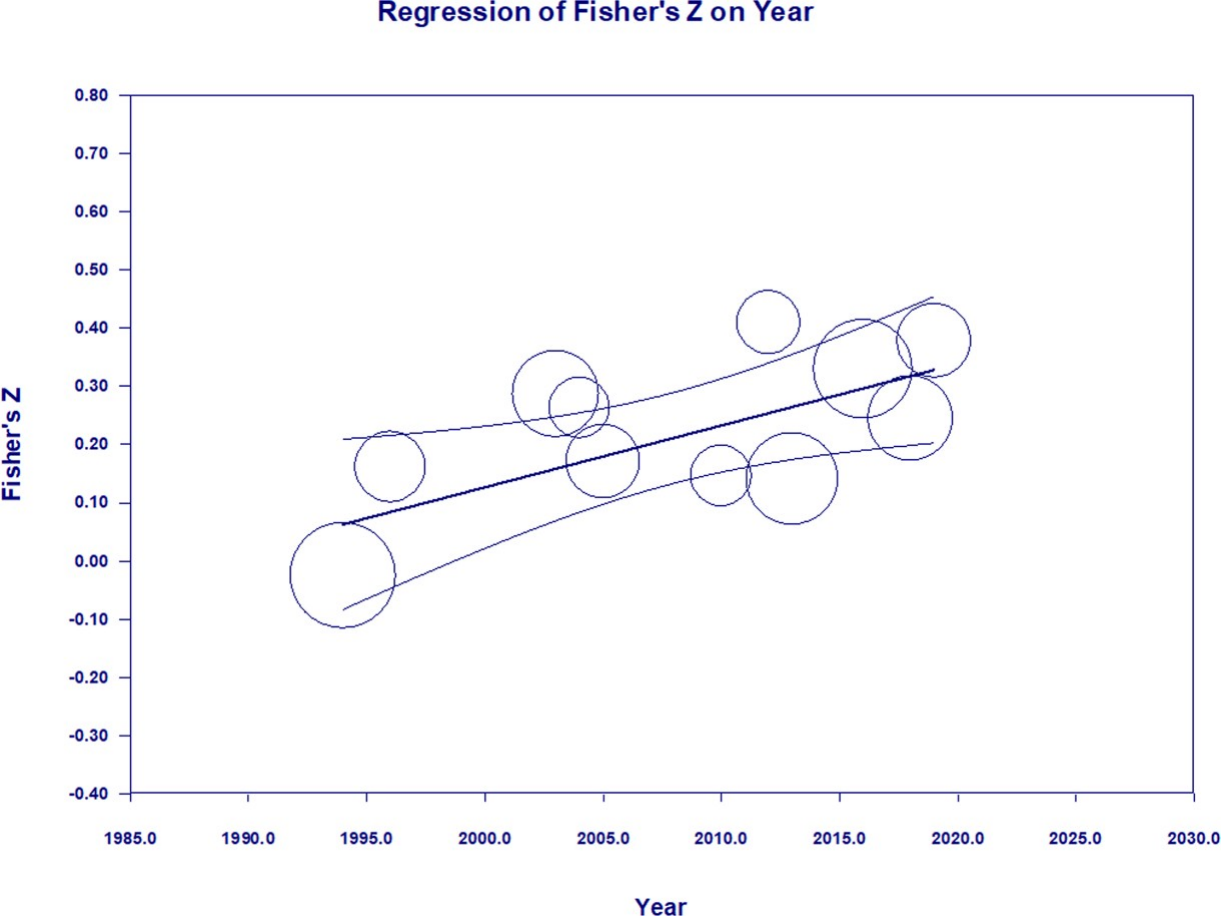
The strength of the relationship between time management and job performance increases over the years.

Moreover, we note that the relationship between time management and job performance (see Fig 2) significantly increases over the years (*B* = .0106, *p* < .01, Q_model_ = 8.52(1), Q_residual_ = 15.54(9), *I*^2^ = 42.08, *R*^2^_analog_ = .75).

### Time management and performance in academic settings

Overall, the effect of time management on performance seems to be slightly higher in academic settings compared to work settings, although the magnitude of the effect remains moderate (see Table 5). Here again, we distinguish between results- and behavior-based performance. Time management’s impact on behavior-based performance seems much higher than on results-based performance—a much wider difference than the one we observed in professional settings. This suggests than results-based performance in academic settings depends less on time management than results-based performance in professional settings. This means that time management is more likely to get people a good performance review at work than a strong GPA in school.

**Table 5:** Time management and performance in academic settings.

Variable	k	N	R	95% CI	Q(df)	*τ*^2^	*τ*^2^(SE)	*I*^*2*^
**Academic Achievement (overall)**	76	30,605	0.262***	0.223–0.300	916.31 (75)	0.029	0.007	91.81
** Results-based performance (overall)**	63	27,225	0.196***	0.160–0.232	535.28 (62)	0.018	0.005	88.41
GPA	57	24,270	0.213***	0.178–0.247	384.48 (56)	0.014	0.004	85.43
Standardized Tests	7	6,270	0.011	-0.053–0.094	33.35 (6)	0.007	0.006	82.01
Test Scores	3	603	0.228***	0.151–0.303	1.21 (2)	0	0.005	0
** Behavior-based performance (overall)**	28	8,186	0.430***	0.365–0.490	310.83 (27)	0.037	0.013	91.31
Procrastination (reverse coded)	14	3,558	0.490***	0.399–0.572	136.62 (13)	0.040	0.020	90.48
Motivation	17	5,805	0.381***	0.302–0.454	178.85 (16)	0.031	0.013	91.05

* p < .05

** p < .01

*** p < .001.

k = number of studies related to the variable | N = total sample size related to the variable.

r = effect size of the correlation between time management and the variable | 95% CI = confidence interval of the effect size.

Q = Cochran’s Q, a measure of between-study heterogeneity | *τ*^2^ = measure of between-study variance | *I*^*2*^ = alternative measure of between-study heterogeneity.

In particular, time management seems to be much more negatively related to procrastination in school than at work. Although we cannot establish causation in all studies, we note that some of them featured experimental designs that established a causal effect of time management on reducing procrastination [90].

Interestingly, time management was linked to all types of results-based performance except for standardized tests. This is perhaps due to the fact that standardized tests tap more into fluid intelligence, a measure of intelligence independent of acquired knowledge [91]. GPA and regular exam scores, in contrast, tap more into crystallized intelligence, which depends mostly on accumulated knowledge. Time management can thus assist students in organizing their time to acquire the knowledge necessary to ace a regular exam; for standardized exams that depend less on knowledge and more on intelligence, however, time management may be less helpful. Evidence from other studies bears this out: middle school students’ IQ predicts standardized achievement tests scores better than self-control while self-control predicts report card grades better than IQ [92]. (For our purposes, we can use self-control as a very rough proxy for time management.) Relatedly, we found no significant relationship between time management and cognitive ability in our meta-analysis (see Table 8).

### Time management and wellbeing

On the whole, time management has a slightly stronger impact on wellbeing than on performance. This is unexpected, considering how the dominant discourse points to time management as a skill for professional career development. Of course, the dominant discourse also frames time management as necessary for wellbeing and stress reduction, but to a much lesser extent. Our finding that time management has a stronger influence on wellbeing in no way negates the importance of time management as a work skill. Rather, this finding challenges the intuitive notion that time management is more effective for work than for other life domains. As further evidence, notice how in Table 6 the effect of time management on *life* satisfaction is 72% stronger than that on *job* satisfaction.

**Table 6:** Time management and wellbeing.

Variable	k	N	r	95% CI	Q(df)	*τ*^2^	*τ*^2^(SE)	*I*^*2*^
**Overall wellbeing**	30	9,905	0.313***	0.244–0.380	395.83 (29)	0.040	0.014	92.67
Job satisfaction	11	2,856	0.248***	0.189–0.305	24.21 (10)	0.006	0.005	58.70
Life satisfaction	9	2,855	0.426***	0.273–0.558	175.86 (8)	0.068	0.038	95.45
Mental health (positive)	2	473	0.556***	0.349–0.711	7.56 (1)	0.031	0.051	86.77
Optimism	2	330	0.305**	0.108–0.479	3.44 (1)	0.016	0.032	70.94
Physical health (positive)	2	567	0.293	-0.002–0.542	13.07 (1)	0.045	0.068	92.35
Positive affect	5	2,725	0.280***	0.186–0.368	18.73 (4)	0.010	0.010	78.65
Self-actualization	1	336	0.280***	0.178–0.376	-	-	-	-
Sense of purpose	1	529	0.351***	0.274–0.424	-	-	-	-
Wellbeing	5	1,447	0.219**	0.092–0.338	22.86 (4)	0.018	0.016	82.50

* p < .05

** p < .01

*** p < .001.

k = number of studies related to the variable | N = total sample size related to the variable.

r = effect size of the correlation between time management and the variable | 95% CI = confidence interval of the effect size.

Q = Cochran’s Q, a measure of between-study heterogeneity | *τ*^2^ = measure of between-study variance | *I*^*2*^ = alternative measure of between-study heterogeneity.

### Time management and distress

Time management seems to allay various forms of distress, although to a lesser extent than it enhances wellbeing. The alleviating effect on psychological distress is particularly strong (*r* = -0.358; see Table 7).

**Table 7:** Time management and distress.

Variable	k	N	r	95% CI	Q(df)	*τ*^2^	*τ*^2^(SE)	*I*^*2*^
**Overall distress**	58	15,387	-0.222***	-0.273 | -0.170	611.57 (57)	0.038	0.010	90.68
** Overall stress**	26	5,621	-0.225***	-0.295 | -0.153	184.49 (25)	0.031	0.012	86.44
Emotional exhaustion	3	213	-0.260***	-0.338 | -0.179	1.86 (2)	0	0.006	0
Stress	17	3,367	-0.286***	-0.390 | -0.176	163.84 (16)	0.05	0.024	90.23
Work-life conflict	9	2,812	-0.163**	-0.277 | -0.043	82.11 (8)	0.031	0.018	90.25
** Overall psychological distress**	34	10,100	-0.254***	-0.315 | -0.190	350.58 (33)	0.034	0.012	90.85
Anxiety	16	6,648	-0.181***	-0.255 | -0.105	140.28 (15)	0.021	0.011	89.30
Depression	2	625	-0.226**	-0.375 | -0.065	-	-	-	-
Psychological distress	10	2,196	-0.358***	-0.447 | -0.263	52.98 (9)	0.023	0.014	83.01
Hopelessness	2	565	-0.218***	-0.296 | -0.138	-	-	-	-
Boredom	5	1,248	-0.310**	-0.507 | -0.081	69.68 (4)	0.070	0.055	94.26
Negative affect	4	2,393	-0.232	-0.451 | 0.014	70.74 (3)	0.061	0.061	95.75
Worry	3	291	-0.191*	-0.355 | -0.016	3.98 (2)	0.012	0.025	49.77
** Physical distress**	7	2,067	-0.204***	-0.264 | -0.142	11.52 (6)	0.003	0.004	47.93

* p < .05

** p < .01

*** p < .001.

k = number of studies related to the variable | N = total sample size related to the variable.

r = effect size of the correlation between time management and the variable | 95% CI = confidence interval of the effect size.

Q = Cochran’s Q, a measure of between-study heterogeneity | *τ*^2^ = measure of between-study variance | *I*^*2*^ = alternative measure of between-study heterogeneity.

That time management has a weaker effect on distress should not be surprising. First, wellbeing and distress are not two poles on opposite ends of a spectrum. Although related, wellbeing and distress are distinct [93]. Thus, there is no reason to expect time management to have a symmetrical effect on wellbeing and distress. Second, and relatedly, the factors that influence wellbeing and distress are also distinct. Specifically, self-efficacy (i.e., seeing oneself as capable) is a distinct predictor of wellbeing while neuroticism and life events in general are distinct predictors of distress [94]. It stands to reason that time management can enhance self-efficacy. (Or, alternatively, that people high in self-efficacy would be more likely to engage in time management, although experimental evidence suggests that time management training makes people feel more in control of their time [89]; it is thus plausible that time management may have a causal effect on self-efficacy. Relatedly, note how time management ability is strongly related to internal locus of control in Table 8) In contrast, time management can do considerably less in the way of tackling neuroticism and dampening the emotional impact of tragic life events. In other words, the factors that affect wellbeing may be much more within the purview of time management than the factors that affect distress. For this reason, time management may be less effective in alleviating distress than in improving wellbeing.

**Table 8:** Time management and individual differences.

Variable	k	N	r	95% CI	Q(df)	*τ*^2^	*τ*^2^(SE)	*I*^*2*^
**Demographics**								
Age	21	7,579	0.032	-0.013–0.076	70.42 (20)	0.007	0.004	71.60
Age (excluding children)	19	6,811	0.048*	0.010–0.086	40.71 (18)	0.004	0.002	55.79
Gender^a^	37	16,044	-0.087***	-0.129 | -0.045	232.40 (36)	0.013	0.005	84.51
Education	3	808	0.019	-0.050–0.088	0.304 (2)	0	0.005	0
Number of children	3	961	0.027	-0.037–0.090	0.247 (2)	0	0.004	0
Marital status ^b^	3	980	0.015	-0.048–0.078	0.548 (2)	0	0.003	0
**Personality**								
Agreeableness	10	4,562	0.169***	0.091–0.244	57.85 (9)	0.013	0.008	84.43
Extraversion	13	5,345	0.102**	0.039–0.164	59.05 (12)	0.010	0.006	79.67
Conscientiousness	15	5,159	0.451***	0.326–0.561	367.16 (14)	0.079	0.041	96.18
Neuroticism	14	5,222	-0.151***	-0.229 | -0.072	94.61 (13)	0.018	0.010	86.26
Openness	11	4,793	0.141**	0.037–0.243	124.17 (10)	0.028	0.016	91.94
**Personal attributes and attitudes**								
Internal locus of control	3	579	0.346***	0.269–0.419	2.16 (2)	0	0.006	7.39
Type A	7	2,388	0.110*	0.017–0.202	31.05 (6)	0.013	0.09	80.67
Self-esteem	3	947	0.346***	0.225–0.456	8.19 (2)	0.010	0.014	75.58
Protestant Work Ethic	3	998	0.026	-0.036–0.088	0.240 (2)	0	0.003	0
Multitasking	5	932	-0.088*	-0.164 | -0.010	5.53 (4)	0.002	0.006	27.66
Cognitive ability	3	1,484	0.015	-0.064–0.094	4.36 (2)	0.003	0.005	54.11
Hours spent studying	6	3,184	0.137**	0.036–0.235	30.08 (5)	0.012	0.011	83.37
Hours spent working	8	3,682	-0.042	-0.159–0.076	64.87 (7)	0.023	0.019	89.21
**Contextual factors**								
Job autonomy	4	751	0.101	-0.060–0.256	8.38 (3)	0.016	0.022	64.23
Role overload	7	1,187	-0.146*	-0.284 | - 0.003	26.59 (6)	0.025	0.023	77.43
Time management training	3	846	0.173*	0.031–0.309	5.92 (2)	0.010	0.016	66.62

* p < .05

** p < .01

*** p < .001.

^a^ Female = 1; Male = 2.

^b^ Single = 1; Married = 2.

k = number of studies related to the variable | N = total sample size related to the variable.

r = effect size of the correlation between time management and the variable | 95% CI = confidence interval of the effect size.

Q = Cochran’s Q, a measure of between-study heterogeneity | *τ*^2^ = measure of between-study variance | *I*^*2*^ = alternative measure of between-study heterogeneity.

### Time management and individual differences

Time management is, overall, less related to individual differences than to other variables.

Age, for instance, hardly correlates with time management (with a relatively high consistency between studies, *I*^*2*^ = 55.79, see Table 8 above).

**Fig 3:**
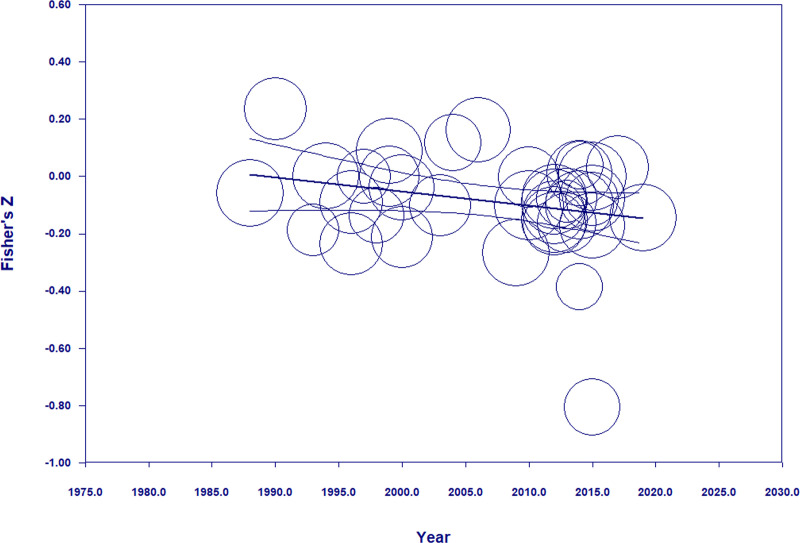
The link between time management ability and gender is getting stronger over the years (lower scores mean stronger skills).

Similarly, gender only tenuously correlates with time management, although in the expected direction: women seem to have stronger time management abilities than men. The very weak association with gender (*r* = -0.087) is particularly surprising given women’s well-documented superior self-regulation skills [95]. That being said, women’s time management abilities seem to grow stronger over the years (*N =* 37, *B* = -.0049, *p* < .05, Q_model_ = 3.89(1), Q_residual_ = 218.42(35), *I*^2^ = 83.98, *R*^2^_analog_ = .03; also see Fig 3 below). More realistically, this increase may not be due to women’s time management abilities getting stronger per se but, rather, to the fact that women now have more *freedom* to manage their time [96].

Other demographic indicators, such as education and number of children, were nonsignificant. Similarly, the relationships between time management and personal attributes and attitudes were either weak or nonsignificant, save for two notable exceptions. First, the link between time management and internal locus of control (i.e., the extent to which people perceive they’re in control of their lives) is quite substantial. This is not surprising, because time management presupposes that people believe they can change their lives. Alternatively, it may be that time management helps people strengthen their internal locus of control, as experimental evidence suggests [89]. Second, the link between time management and self-esteem is equally substantial. Here again, one can make the argument either way: people with high self-esteem might be confident enough to manage their time or, conversely, time management may boost self-esteem. The two options are not mutually exclusive: people with internal loci of control and high self-esteem levels can feel even more in control of their lives and better about themselves through time management.

We also note a very weak but statistically significant *negative* association between time management and multitasking. It has almost become commonsense that multitasking does not lead to performance [97]. As a result, people with stronger time management skills might deliberately steer clear of this notoriously ineffective strategy.

In addition, time management was mildly related to hours spent studying but not hours spent working. (These variables cover only student samples working part- or full-time and thus do not apply to non-student populations.) This is consistent with time-use studies revealing that teenagers and young adults spend less time working and more time studying [98]. Students who manage their time likely have well-defined intentions, and trends suggest those intentions will target education over work because, it is hoped, education offers larger payoffs over the long-term [99].

In terms of contextual factors, time management does not correlate significantly with job autonomy. This is surprising, as we expected autonomy to be a prerequisite for time management (i.e., you can’t manage time if you don’t have the freedom to). Nevertheless, qualitative studies have shown how even in environments that afford little autonomy (e.g., restaurants), workers can carve out pockets of time freedom to momentarily cut loose [100]. Thus, time management behaviors may flourish even in the most stymying settings. In addition, the fact that time management is associated with less role overload and previous attendance of time management training programs makes sense: time management can mitigate the effect of heavy workloads and time management training, presumably, improves time management skills.

Finally, time management is linked to all personality traits. Moreover, previous reviews of the literature have commented on the link between time management and conscientiousness in particular [32]. What our study reveals is the substantial magnitude of the effect (*r* = 0.451). The relationship is not surprising: conscientiousness entails orderliness and organization, which overlap significantly with time management. That time management correlates so strongly with personality (and so little with other individual differences) lends credence to the dispositional view of time management [101–103]. However, this finding should not be taken to mean that time management is a highly inheritable, fixed ability. Having a “you either have it or you don’t” view of time management is not only counterproductive [104] but also runs counter to evidence showing that time management training does, in fact, help people manage their time better.

## Discussion

Does time management work? It seems so. Time management has a moderate influence on job performance, academic achievement, and wellbeing. These three outcomes play an important role in people’s lives. Doing a good job at work, getting top grades in school, and nurturing psychological wellbeing contribute to a life well lived. Widespread exhortations to get better at time management are thus not unfounded: the importance of time management is hard to overstate.

### Contributions

Beyond answering the question of whether time management works, this study contributes to the literature in three major ways. First, we quantify the impact of time management on several outcomes. We thus not only address the question of whether time management works, but also, and importantly, gauge *to what extent* time management works. Indeed, our meta-analysis covers 53,957 participants, which allows for a much more precise, quantified assessment of time management effectiveness compared to qualitative reviews.

Second, this meta-analysis systematically assesses relationships between time management and a host of individual differences and contextual factors. This helps us draw a more accurate portrait of potential antecedents of higher (or lower) scores on time management measures.

Third, our findings challenge intuitive ideas concerning what time management is for. Specifically, we found that time management enhances wellbeing—and in particular life satisfaction—to a greater extent than it does various types of performance. This runs against the popular belief that time management primarily helps people perform better and that wellbeing is simply a byproduct of better performance. Of course, it may be that wellbeing gains, even if higher than performance gains, hinge on performance; that is to say, people may need to perform better as a prerequisite to feeling happier. But this argument doesn’t jibe with experiments showing that even in the absence of performance gains, time management interventions do increase wellbeing [89]. This argument also founders in the face of evidence linking time management with wellbeing among the unemployed [105], unemployment being an environment where performance plays a negligible role, if any. As such, this meta-analysis lends support to definitions of time management that are not work- or performance-centric.

### Future research and limitations

This meta-analysis questions whether time management should be seen chiefly as a performance device. Our questioning is neither novel nor subversive: historically people have managed time for other reasons than efficiency, such as spiritual devotion and philosophical contemplation [72, 106, 107]. It is only with relatively recent events, such as the Industrial Revolution and waves of corporate downsizing, that time management has become synonymous with productivity [43, 65]. We hope future research will widen its scope and look more into outcomes other than performance, such as developing a sense of meaning in life [108]. One of the earliest time management studies, for instance, explored how time management relates to having a sense of purpose [73]. However, very few studies followed suit since. Time management thus stands to become a richer, more inclusive research area by investigating a wider array of outcomes.

In addition, despite the encouraging findings of this meta-analysis we must refrain from seeing time management as a panacea. Though time management can make people’s lives better, it is not clear how easy it is for people to learn how to manage their time adequately. More importantly, being “good” at time management is often a function of income, education, and various types of privilege [42, 43, 46, 109]. The hackneyed maxim that “you have as many hours in a day as Beyoncé,” for instance, blames people for their “poor” time management in pointing out that successful people have just as much time but still manage to get ahead. Yet this ill-conceived maxim glosses over the fact that Beyoncé and her ilk do, in a sense, have more hours in a day than average people who can’t afford a nanny, chauffeur, in-house chefs, and a bevy of personal assistants. Future research should thus look into ways to make time management more accessible.

Furthermore, this meta-analysis rests on the assumption that time management training programs do enhance people’s time management skills. Previous reviews have noted the opacity surrounding time management interventions—studies often don’t explain what, exactly, is taught in time management training seminars [18]. As a result, comparing the effect of different interventions might come down to comparing apples and oranges. (This might partly account for the high heterogeneity between studies.) We hope that our definition of time management will spur future research into crafting more consistent, valid, and generalizable interventions that will allow for more meaningful comparisons.

Finally, most time management studies are cross-sectional. Yet it is very likely that the effect of time management compounds over time. If time management can help students get better grades, for instance, those grades can lead to better jobs down the line [110]. Crucially, learning a skill takes time, and if time management helps people make the time to learn a skill, then time management stands to dramatically enrich people’s lives. For this reason, longitudinal studies can track different cohorts to see how time management affects people’s lives over time. We expect that developing time management skills early on in life can create a compound effect whereby people acquire a variety of other skills thanks to their ability to make time.

## Conclusion

Overall, this study offers the most comprehensive, precise, and fine-grained assessment of time management to date. We address the longstanding debate over whether time management influences job performance in revealing a positive, albeit moderate effect. Interestingly, we found that time management impacts wellbeing—and in particular life satisfaction—to a greater extent than performance. That means time management may be primarily a wellbeing enhancer, rather than a performance booster. Furthermore, individual and external factors played a minor role in time management, although this does not necessarily mean that time management’s effectiveness is universal. Rather, we need more research that focuses on the internal and external variables that affect time management outcomes. We hope this study will tantalize future research and guide practitioners in their attempt to make better use of their time.

## Supporting information

S1 Checklist PRISMA 2009 checklist. (DOC)

S1 File Funnel plots. (PDF)

S2 File Dataset. (XLSX)
