# Supplementary material for: Does time management work? A meta-analysis
Source: PLoS One. 2021 Jan 11;16(1):e0245066. doi: 10.1371/journal.pone.0245066 (PMC7799745; doi:10.1371/journal.pone.0245066)

Supporting File S1. Funnel Plots.

Funnel plot: time management and distress. Funnel plot of standard error by Fisher's Z.

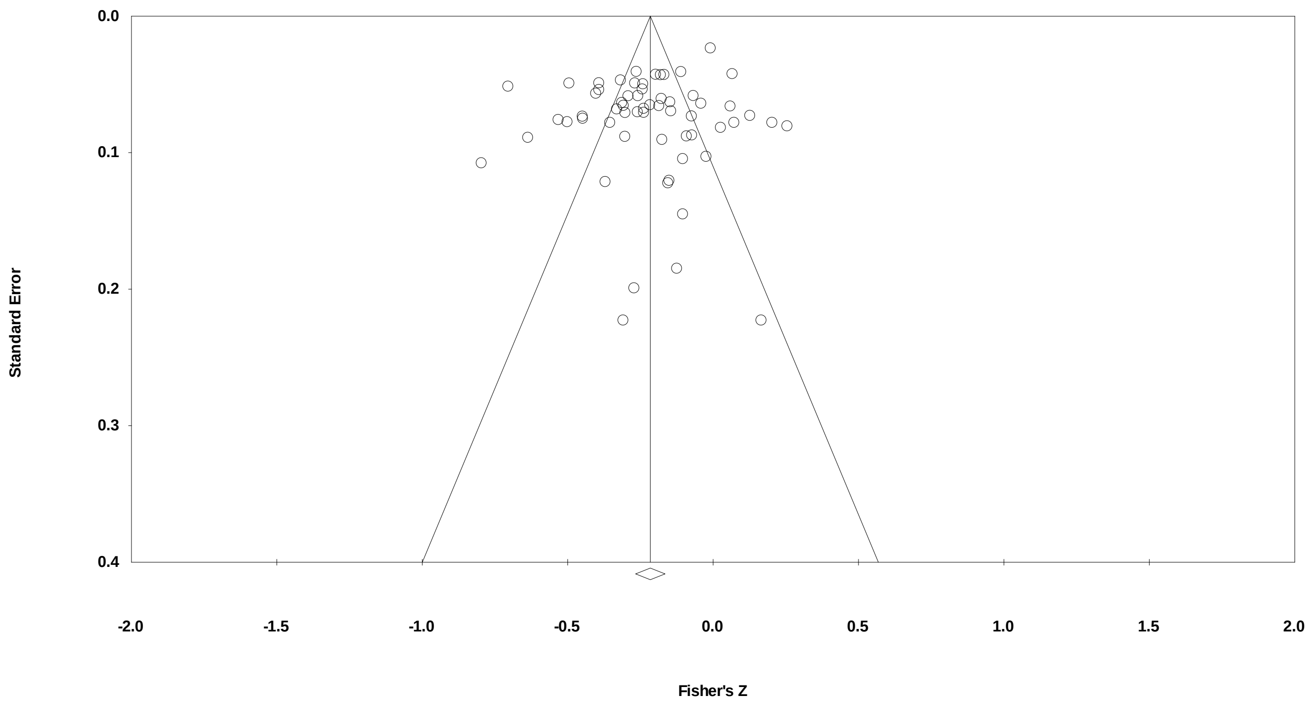

Funnel plot: time management and wellbeing. Funnel plot of standard error by Fisher's Z.

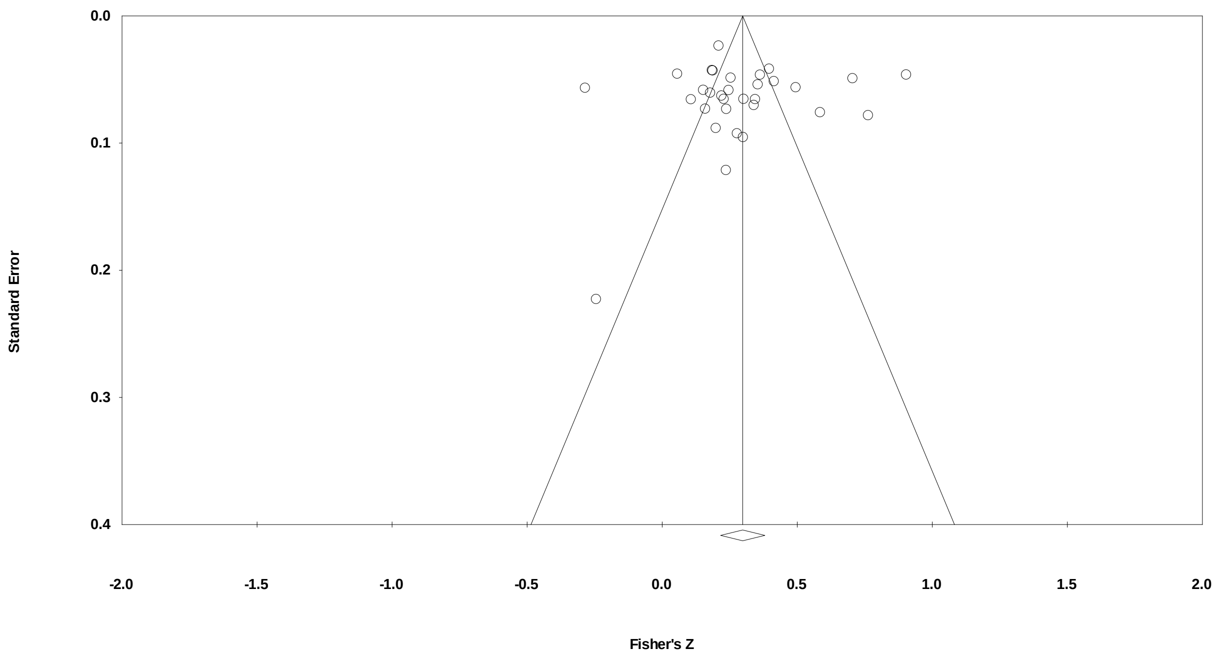

Funnel plot: time management and academic achievement. Funnel plot of standard error by Fisher’s Z.

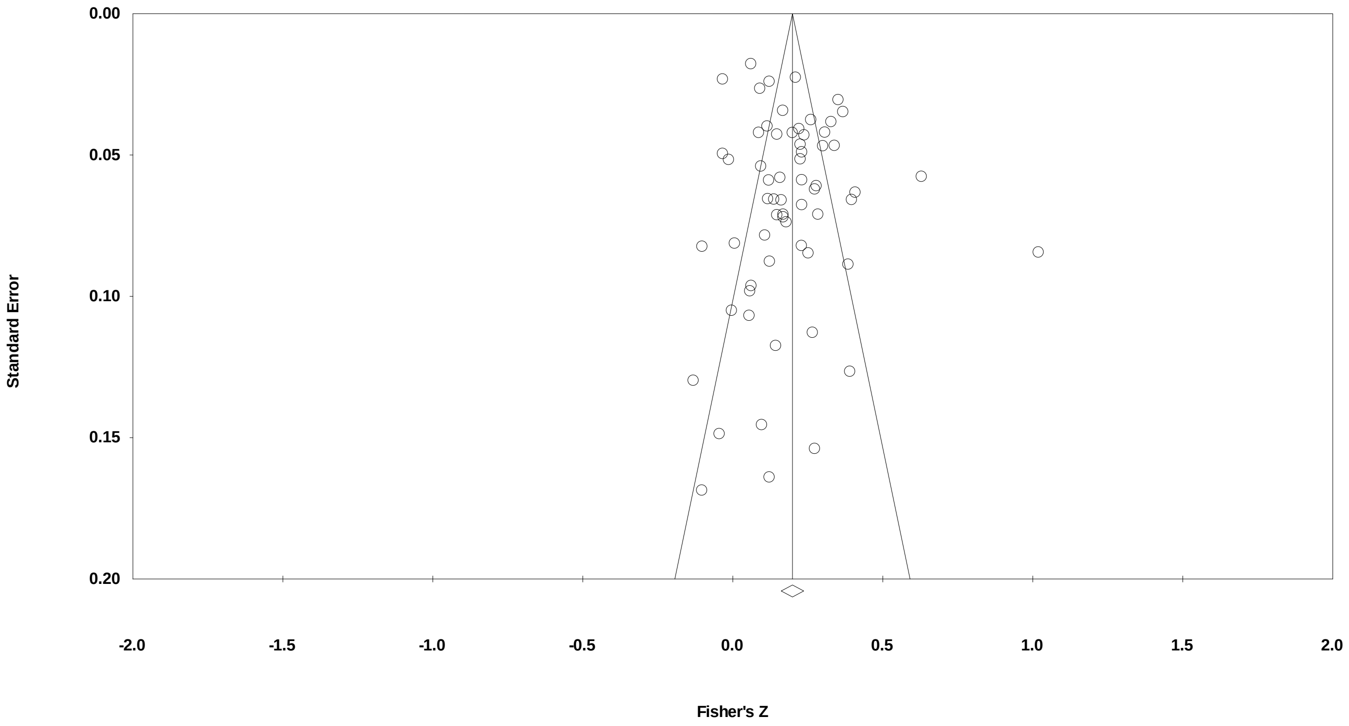

Funnel plot: time management and job performance. Funnel plot of standard error by Fisher’s Z.

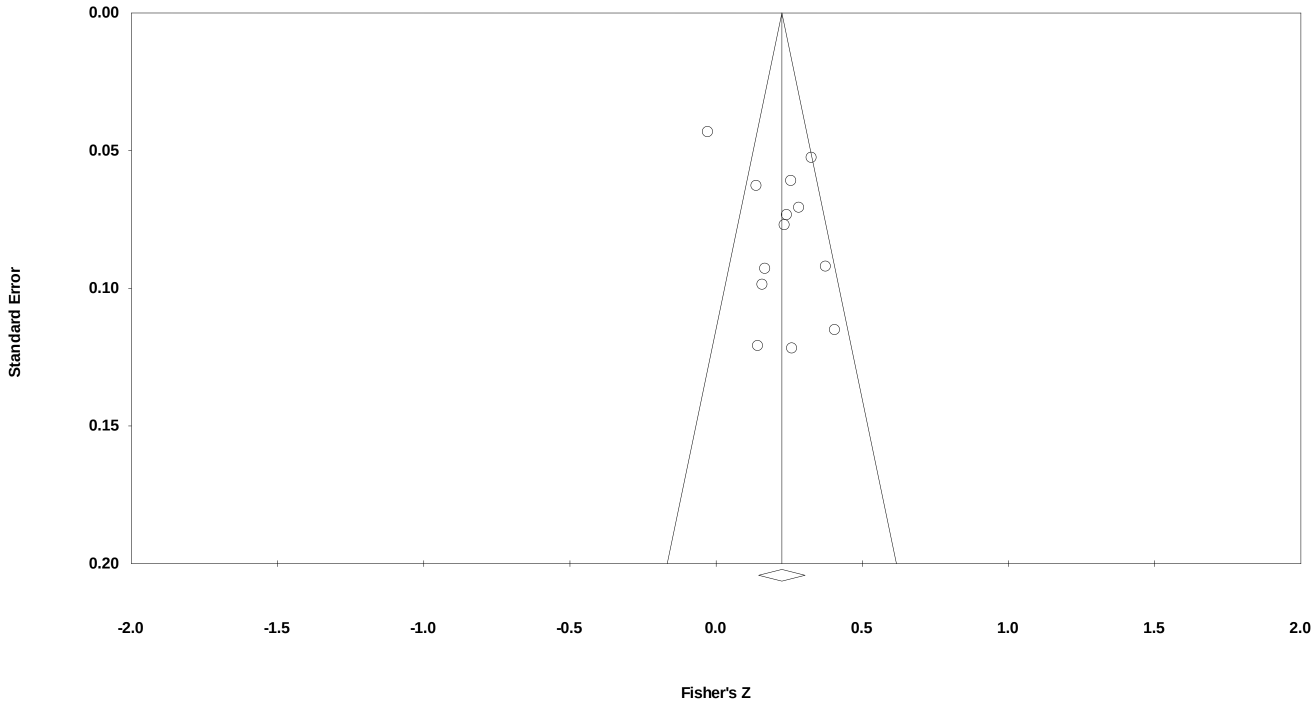

Supplement: S1 File — (PDF) [file pone.0245066.s002.pdf]
